# Responsible research impact: Ethics for making a difference

**DOI:** 10.12688/openreseurope.19649.1

**Published:** 2025-03-31

**Authors:** Eric Allen Jensen, Mark S. Reed, James Daybell, Louise Rutt, Aaron M. Jensen, Gabriella Arrigoni, Marta Ballesteros, Sadiq Bhanbhro, Bethann G. Merkle, Caitlin Hafferty, Philly Iglehart, Sawsan Khuri, Andrzej Klimczuk, Ian D. Marder, Daniel Milosavljevic, Josmel Pacheco-Mendoza, Ursula Pool, Simon Robinson, Lindsay C. Stringer, Steve Taylor, Anne H. Toomey, Daniela Martin, Lisa Louise Taylor-Sayles, Andrew N. Makohon-George, Russell T. Rodrigo

**Affiliations:** 1 Institute for Methods Innovation, Dublin, D02 XE80, Ireland; 2 Natural Capital Challenge Centre, Scotland's Rural College (SRUC), Edinburgh, EH9 3JG, UK; 3 Faculty of Arts, Humanities and Business, University of Plymouth, Plymouth, PL4 8AA, UK; 4 Northumbria University, Newcastle upon Tyne, England, NE1 8ST, UK; 5 Spanish Institute of Oceanography (IEO-CSIC), Madrid, 28002, Spain; 6 Centre for Applied Health and Social Care Research, Sheffield Hallam University, Sheffield, England, UK; 7 Department of Zoology and Physiology, University of Wyoming, Laramie, Wyoming, WY 82071, USA; 8 University of Oxford, Oxford, England, OX1 2JD, UK; 9 Plymouth Marjon University, Plymouth, England, PL6 8BH, UK; 10 Collaborative Capacities, Exeter, EX2 4DG, UK; 11 SGH Warsaw School of Economics, Warsaw, Poland; 12 Maynooth University, Maynooth, County Kildare, Ireland; 13 Manaaki Whenua - Landcare Research, Lincoln, New Zealand; 14 Bibliometrics, Evidence Evaluation and Systematic Review Group (BEERS), Human Medicine Career, Universidad Cientifica del Sur, Lima, Peru; 15 Healthy and Sustainable Settings Unit, University of Central Lancashire, Preston, England, PR1 2HE, UK; 16 Swansea University, Swansea, Wales, UK; 17 Department of Environment and Geography, University of York, York, England, UK; 18 York Environmental Sustainability Institute, University of York, York, England, UK; 19 AngelWings Ltd, Dunedin, New Zealand; 20 Pace University, New York, New York, USA; 21 College of the Redwoods, Eureka, California, CA 95501, USA; 22 Bangkok University International, Bangkok, 12120, Thailand

**Keywords:** ethical guidelines, research ethics, research integrity, public engagement, research impact, policy engagement, stakeholder analysis, community engagement

## Abstract

The need for ethical guidelines that support and empower researchers who aim to enhance the societal impact of research has become critical. Recognizing the growing emphasis on research impact by governments and funding bodies worldwide, this article investigates the often overlooked ethical dimensions of generating and evaluating research impact. We focus on ethical issues and practices that are specific to the process of intentionally working to develop societal impacts from research. We highlight the complexities and ethical dilemmas encountered when researchers engage with non-academic groups, such as policymakers, industries, and local communities. Through a combination of literature review and insights from participatory workshops, the article identifies key issues and offers a new ethical framework for responsible research impact. This framework aims to guide researchers and institutions through the process of limiting potential harm while delivering societal benefits in a way that is realistic and balanced. The aim is to establish ethical practices for engagement and impact, without making the process so onerous that researchers are less likely to undertake such activities. The article concludes with actionable recommendations for policymakers, research funders, research performing organizations, institutional review boards and/or ethics committees, and individual researchers. Making use of such recommendations can foster an ethically responsible approach to research impact across academic disciplines.

## Plain language summary

Researchers are increasingly expected to produce work that benefits society. However, when trying to make research useful for the broader public—by working with policymakers, industries, or communities—there can be complex ethical challenges. This article argues that we need guidelines to help researchers handle these challenges in a responsible and ethical way. Drawing on both existing scholarship and hands-on discussions with researchers, the article identifies key ethical issues involved in creating and evaluating social impact from research. These include questions about fairness (who benefits and who might be harmed), consent (whether all participants are fully informed and agree), transparency (how and why research is shared), and accountability (making sure any negative impacts are minimized). To help improve ethical practices aimed at benefiting society, the authors propose a new framework. This framework is designed to help researchers and the institutions that support them think through potential ethical problems in their efforts to create positive change. It also offers practical recommendations for policymakers, research ethics boards and individual researchers. By following these guidelines, researchers can better protect the interests of the people they work with and ensure that their research has a meaningful and responsible impact on society.

## 1. Introduction

Globally, governments, funders, universities, and researchers are increasingly investing in the societal impact of research, seeing it as an unquestioned good (e.g., Chubb & Watermeyer, 2017; Smith & Smith, 2020a). There is growing normative pressure to demonstrate the impact of research as a value of publicly funded research investments ( Oancea, 2019). There is also rising consensus that it is critical to ensure that the benefits of research are shared with all segments of society, not just a privileged few. (For example, this is part of the UN human rights framework and has been specifically highlighted in UNESCO’s recently reinvigorated Recommendation on Science and Scientific Researchers ( Jensen, 2020)). It is argued that those who contribute to research, whether directly (e.g., as participants) or indirectly (e.g., as taxpayers), have the right to benefit in some tangible way from the work that is done (e.g., Antoni & Beer, 2019). While it may not be realistic to expect impact, at least in the short to medium term, from non-applied research, funders and governments increasingly expect researchers to plan for impact and provide examples of the benefits that arise from at least some of the research that is funded (e.g., via impact case studies in assessments like the UK’s Research Excellence Framework (REF), Hong Kong China’s Research Assessment Exercise, and the focus on broader impacts in U.S. National Science Foundation funding ( Bornmann & Haunschild, 2019; Nabi, 2018). In addition to this, there are growing concerns about negative unintended consequences arising from research (e.g., Derrick *et al.*, 2018; Reed & Fazey, 2021) and the effects of research assessments on researcher behavior ( Watermeyer *et al.*, 2022) and research culture ( Reed & Fazey, 2021). This places a burden on individual researchers to undertake ethically responsible impact, and it is thus vital that such researchers, as well as funders, governments, and other societal actors, are clear on what constitutes ethical or unethical research impact processes and outcomes.

Developing societal impact from and through research is fraught with ethical quandaries related to who is engaged, how, and by what criteria research is deemed impactful (e.g., Jensen, 2022). Yet, the need to systematically address the ethics involved in this type of research impact generation has been neglected ( Bærøe *et al.*, 2022). Ethical oversight from research funders and governments on this topic is rare. Furthermore, there have been no system-wide practical interventions to advance ethical research impact policy and practice, where negative impacts can be predicted and prevented, as well as mitigated in real time. The gap in coverage of research impact ethics has left expectations unclear and decision-making inconsistent. This ambiguity has led to calls for “conversations and tools that allow us to meaningfully consider the ethics of research impact” ( Smith & Smith, 2020b, p. 203). Thus, urgent action is required to self-regulate, set robust, practical, and long-lasting standards and support collective action for the ethics of research impact at the level of the individual researchers, communities of practice, Higher Educational Institutions (HEIs) and other academic organizations and professional societies.

Ethical in this context means striving to avoid harm while maximizing long-term benefits, with particular attention to the needs and aspirations of marginalized, vulnerable/at-risk, and disempowered populations. We formulated the main research question as follows: What key features should be included in an ethical framework guiding researchers’ societal and environmental impacts? Based on this research question, we set three main objectives: (1) identify the main aspects of research impact ethics; (2) provide an analytic overview of existing guidelines on ethical research impact; and (3) formulate practical recommendations on ethics of research impact for international or national policymakers, institutional review boards and/or ethics committees, and individual researchers.

We identify a range of existing initiatives developed internationally aimed at promoting ethical impact, including various guidelines, training programs, professional networks, and toolkits, as well as published examples of different practices adopted by researchers, research management professionals, and institutions to engage with the ethics of research impact. Additionally, we delve into key gaps identified during participatory workshops convened on this subject. Building upon the insights gained from both the literature review and the workshop discussions, we propose a new ethical framework to support ethical research impact across the disciplinary spectrum (while steering clear of debates relating to the ethics of research more broadly).

In this article, we focus on ethical issues and practices tied to the process of intentionally working to develop ethical impacts from research. This process often involves different forms of engagement and other means of pursuing beneficial societal outcomes. Throughout this article, we refer to this broad range of activities ultimately aimed at developing benefits for society as *research impact*. The framework we present is intended for responsible research impact practice and to inform future capacity-building initiatives, aiming to support efforts to embed good practices within institutional cultures (e.g., Broder *et al.*, 2024; Jensen *et al.*, 2021). This article is aimed at informing those with a degree of autonomy in, or a remit for, determining how research impact will be designed, delivered, measured, and evaluated, including researchers, professional services staff, consultants, industry and community leaders, and others supporting the generation of impact from research.

## 2. Methods

We used two methods to develop our proposed framework to foster ethical research impacts: (1) a narrative literature review ( Baumeister & Leary, 1997) to identify an initial set of issues to be addressed based on existing research and (2) a set of online participatory international workshops that gathered insights on relevant issues, guidelines, and recommended good practices from those who responded to an open, public invitation to contribute as co-authors to capture and give credit to their knowledge fully. Each of these methods was employed iteratively throughout the research process.

### 2.1. Initial review of literature and initiatives

We reviewed a range of existing initiatives nationally and internationally that aimed at promoting ethical impact, including various guidelines, training programs, networks, and toolkits, as well as published examples of different practices adopted by researchers and institutions to engage with the ethics of impact. The review of literature and initiatives was approached with an exploratory, expert-based, narrative review method ( Baumeister & Leary, 1997). This approach was needed to account for the diverse range of disciplines contributing to the research impact field and the varied terminology used across the full spectrum of research, from the arts and humanities to medicine and physics. A narrative review approach was chosen due to its suitability for exploratory scenarios where specific interventions or outcomes could not be readily identified ( Greenhalgh *et al.*, 2018). For example, disciplines and topic areas considered for this exhaustive review included higher education scholarship, public and community engagement, open research, implementation science, research ethics, research and impact culture, practical theology, sociology of work, participatory action research, design research, responsible research and innovation, research on research, community and economic regeneration, social responsibility, organizational studies, peace and conflict studies, international development, co-production, resilience, knowledge mobilization, evaluation, public policy ethics, Indigenous studies, applied philosophy, and applied anthropology.

### 2.2. Online workshops

Using an open authorship model, contributions were then invited from across and beyond the research sector (following the CRediT authorship scheme; https://credit.niso.org) via online workshops. This approach was necessary to ensure a diverse, multi-authored article covering the broad range of disciplines involved. Indeed, we were aware that addressing the scope of ethical considerations was unlikely to be manageable if conducted by a single researcher or even by a small multidisciplinary team. The review encompassed both peer-reviewed and grey literature, identifying relevant initiatives at various organizational levels (regional, national, and international).

Building on the existing literature, a set of two online workshops were run in 2024 that involved over 300 participants from Australia, Canada, Falkland Islands, Greece, Ireland, Italy, Netherlands, New Zealand, Peru, Poland, Portugal, Serbia, Spain, Sweden, Switzerland, United Kingdom, and the United States. The workshops used the metaplan technique ( Reed, 2018) via Miro boards (a digital collaboration platform; https://miro.com/) to elicit and then thematically group key issues emerging from the review work (workshop 1) and to refine ethical principles and unpack implications for researchers, research institutions, and funders (workshop 2). An open call was made for each workshop and circulated through email lists and social media platforms. Explicit invitations via email and social media were also targeted at categories of participants based on the issues identified in the literature, aiming to ensure representation from a wide range of relevant parties, including academic (researchers, research managers, and professional services staff) and non-academic groups (e.g., professional associations and research funders).

Written informed consent was sought, and personal data were managed in line with the European Union’s General Data Protection Regulation (GDPR). The workshop facilitators kept detailed notes, and detailed written commentary was elicited from participants using online collaborative tools (i.e., Miro) for integration into the article as part of our open authorship approach. The workshops were designed and led by experienced facilitators, Reed and Jensen.

The first collaborative workshop built on the initial literature review and focused on scoping the article, identifying additional literature sources, and identifying resources that could be included in this article. A thematic analysis of sources identified in the literature was used to propose an initial ethical framework (which included seven principles and an initial description) for discussion at the second workshop. This led to the re-organization and refinement of principles and their description and the identification of lessons for researchers and institutions linked to each principle. This was further refined by workshop participants as co-authors of this article, producing six principles for ethical research impact.

## 3. Research impact ethics scope

The potential scope for a discussion of ethics in research impact is nearly endless. Given the overwhelming range of issues and practices that could be considered relevant, for practical reasons, we have narrowed our focus to the most pressing subset of issues, that is, activities or approaches that would constitute “unethical practice”. Therefore, we address what *needs* to be done to reduce the risk that research impact work is *unethical* to the extent researchers can control processes and outcomes. An allied central aim was to think about ways of embedding ethical considerations into institutional impact and research cultures within research institutions.

Given this scope, we hope future work will address the larger universe of positive steps that could make research impact work more ethical. For example, many researchers feel an ethical responsibility to develop societal benefits from publicly funded research ( Jensen & Holliman, 2016). Such an implicit ethical mandate to deliver impact is outside the scope of this article.

At the same time, reducing research impact ethics to a bureaucratic process of gaining approval from a committee or institutional review board (the current framework governing research ethics in the Global North) casts the net too narrowly and risks unnecessarily constraining innovative approaches and academic autonomy. Indeed, we affirm that impact ethics “includes considerations of the relationships between researchers and non-academic [relevant parties] and the values that underpin these endeavors” ( Bayley & Phipps, 2023, p. 8). Therefore, engagement and participation methods are in scope insofar as they are ultimately aimed at ethical research impact.

There are, of course, broader questions regarding the ethics of how research is assessed and funded and how assessors and funders define, incentivize, measure, and operationalize impact ( Bornmann, 2012). These questions, though valid and important, are also outside our scope. Similarly, although research impact is clearly, to some degree, affected by the research ethics underpinning the production of the research, our focus is on the ethics of *research impact* rather than research ethics. Unlike research impact ethics, there is an extensive literature, practical guidelines, and existing institutional review and parameters overseeing research ethics, research integrity, the implications of research for particular topics, and open science. Thus, to maintain our focus on the ethics of research impact, the following upstream topics were excluded from this review:

1. Designing, developing, and deploying research studies ethically and complying with processes of gaining institutional research ethics approval, including obtaining informed consent to participate in research, ethical use and management of research data, maintaining privacy (such as GDPR in the EU), and protecting research participants (except where the same people are involved in impact generation activities).
2. Conflicts of interest affecting research topic selection, methods, and results (including where these involve impact-oriented co-production, for example).
3. Justice and equity issues relevant to research processes, including socially inclusive research participation and the empowerment of marginalized people during the research process.
4. Accurate reporting of research findings, data, and methodology, as well as avoiding questionable research practices and fraud.
5. Transparency, openness, and reflexivity of the researcher during the research process.
6. Issues affecting research careers, precarity, and diversity in research fields and teams.
7. Open science principles and practices.
8. Downstream societal implications of creating new knowledge writ large.
9. Issues around background intellectual property and copyright.

At the same time, the line between the ethics of research on the one hand and research impact on the other is often artificial. Maintaining these distinctions becomes largely impossible in co-production, participatory methods, and other types of research practice that integrate engagement, impact, and research data collection. While, in establishing the scope of this article, we aimed to maintain this distinction as best we could as a useful conceptual device, we discuss later how the more that impact is embedded within research, the more progressive the approach to impact ethics may become.

## 4. Results: Key aspects of research impact ethics

In the various policy and funding initiatives bolstering research impact, there is a prevailing normative assumption that the impact will and should be positive. Indeed, “the possibility that research impact could be negative, or even open to interpretation, is almost never articulated” ( Smith & Smith, 2020a, p. 14, writing about the UK research assessment context, even though the guidelines specifically refer to potential negative impacts). This normative assumption is built into definitions of impact from research funders and research assessment bodies in the UK and in other countries that conduct national assessments of impact, such as the Netherlands, Italy, Finland, Poland, Australia, New Zealand, and the United States. However, ethical approaches to research impact require going beyond the concept of demonstrable impact to consider perceptions of impact processes and results, which are inherently subjective and depend on the context and values of relevant parties (e.g., Reed *et al.*, 2025).

In this light, it becomes important to consider how and why research impact efforts might give rise to harmful, unintended consequences that may differ between groups and shift over time. These harmful results are sometimes called negative impacts or “grimpacts” ( Derrick *et al.*, 2018). Before judging impact results, we must clarify the principles defining “which ways of pursuing impact are acceptable and which are not” ( Bærøe *et al.*, 2022, p. 7). We reviewed relevant ethical issues that apply to intentionally developed research impacts. These may include research impact strategy, planning, risk management, inclusive engagement, involvement and participation, and follow-up and continuous evaluation processes that bolster ethical impact and mitigate negative impact. We discuss the results of our review of potential issues, then identify approaches and considerations that can help to achieve *ethical* research impacts.

### 4.1. Research impact strategy, design, and planning

Research assessments and funding bodies often seek evidence of beneficial research impacts. However, there is growing awareness within the global research community of the responsibility of researchers and their institutions to not only avoid but also manage and mitigate unintended harmful impacts arising from their attempts to deliver societal benefits (e.g., Reed & Rudman, 2023; Reed *et al.*, 2024). In addition to harms resulting from problematic processes for developing impact (e.g., Asase *et al.*, 2022; Canfield *et al.*, 2020; Kimmerer & Artelle, 2024), unintended harmful results can stem from unforeseen and unforeseeable circumstances, illustrating the indirect, non-linear, and intricate nature of research impact generation ( Broder *et al.*, 2024; Crawford, 2020; Posner & Cvitanovic, 2019; Toomey, 2024). To maximize beneficial results and minimize harmful outcomes, impact ethics must be embedded throughout the entire process of developing research impact, starting with impact design and planning for engagement and impact before and during the impact funding stage (where applicable). This should also apply to engagement and impact activities arising from secondary research, where open data are re-analyzed or synthesized to generate recommendations or, indeed, for any other impact uses that were not intended during the initial data collection process.

***4.1.1 Ethics of care and “do no harm”***

Ethical research impact requires, at minimum, receptiveness to the perspectives of the intended beneficiaries. A positive example of this is the UK research project called “Nursing Narratives: Racism and the Pandemic”. The project aimed to benefit UK-based nurses from ethnic minority backgrounds working for the National Health Service. Being responsive to the perspectives of such nurses is important for developing ethical research impact from the project.

Antoni and Beer (2019) propose an ethical framework for research impact based on the concept of “care” inspired by feminist theory. They argue that research impact strategy should be driven by what is genuinely in the best interests of those it is ostensibly aimed at helping, not just what is beneficial to a research institution or a funder. In other words, research impact should be *responsive* to the perspectives of those from outside academia, particularly the intended users and beneficiaries of research findings. Being responsive is a moral imperative and is related to the notion of reciprocity identified in many Indigenous research methods frameworks. Ethical, responsive research impacts require a radical form of empathy that recognizes that we cannot assume that the needs of others conform to our views about what is a desirable outcome. This framework suggests that it is ethically problematic for researchers to push their own impact agendas based solely on their own assumptions about what constitutes good or bad outcomes for affected people. The implication is that co-design, co-creation (including co-production), and co-assessment with those who are potential “beneficiaries” and “victims” (those who might be negatively affected should be fundamental to research impact practice and evaluation.

Darby (2017) argued that there may be an ethical duty to use participatory methods, where current and future beneficiaries/victims of impact are enlisted in impact processes. Reed and Fazey (2021) take this a step further to argue that co-production is integral to the development of “third generation” impact cultures, which, they describe as seeking “to examine, and where necessary question, the assumptions driving the systems that both generate and apply knowledge, asking who generates what knowledge for whom, for what purpose, and why” ( Reed & Fazey, 2021, Background section, para. 5). In contrast to more individualistic and corporate impact cultures, they argue for a move towards more co-productive impact cultures, in which “impact goals are co-produced through active relationship and dialogue with stakeholders as a primary consideration in research” ( Reed & Fazey, 2021, Discussion section, bullet point 4).

In addition to considering the use of participatory methods, ethical duties also require us to scrutinize research impact processes that deliver mixed beneficial and harmful results. For example, research that yields economic or health benefits for society may also employ questionable ethical practices, resulting in significant environmental harm, animal welfare, or even human rights violations. The history of medical research is replete with examples of this kind of mixed outcome (e.g., Warren *et al.*, 2020). Thus, the likely and potential adverse outcomes and risks of research impacts must be explicitly assessed and weighed against realistic expectations of positive outcomes across multiple temporal scales. Proactive weighing of likely outcomes should encompass the opportunity costs associated with investing scarce resources in a research impact initiative compared to alternative interventions using those funds.

Thus, an obvious principle for ethical research impact is “do no harm”. That is, we must all try to avoid exacerbating environmental damage, aggravating social divisions, increasing inequities in health and justice, or further diminishing the power of already marginalized or vulnerable people. Such an approach should help researchers avoid harming the communities and environments they aim to benefit. Similarly, proactive attention to the ethics of research impact can help researchers avoid contributing to declines in public trust or support for the research itself.

However, undertaking this level of self-scrutiny or ethical soul-searching for every research impact intervention could be highly resource intensive. The scope of analysis would be so complex and demanding that few would do it. A light-touch process is needed to encourage due consideration of the implications of proposed and existing research impact activities without demanding a prohibitive investment of time and effort.

In the same spirit, Antoni and Beer (2019) also note the importance of self-care; that is, for those working in research, it is important to consider personal needs and values during the process (e.g., Merkle *et al.*, 2022) acknowledging the emotional and mental strain accompanying such work (e.g., Chubb & Watermeyer, 2017). People engaged in impact-driven activities often grapple with complex and sensitive topics, for example, combating poverty, reducing or preventing terrorism, tackling racism, and improving working conditions in hazardous work environments. Dealing with such topics can weigh heavily on those involved in impact work, perhaps especially where impact can be frustratingly difficult to achieve (e.g., Cordaro, 2020; Velez-Cruz, 2020). These challenges make self-care ( Kumar & Cavallaro 2018; Richard & Shea, 2011) an essential aspect of professional practice — both for one’s own good ( Jiang *et al.*, 2021; Lakshmin, 2023; Tarabochia *et al.*, 2022) and for the good of the people who might be affected by their work ( Nicol & Yee, 2017; Wyatt & Ampadu, 2022). By clarifying one’s own values and relationship to research impacts (e.g., Merkle *et al.*, 2022), setting clear boundaries, and seeking support, those seeking to develop impact can not only safeguard their personal well-being but also help to ensure the long-term sustainability and effectiveness of their efforts Individual efforts by researchers need to be underpinned by enabling and rewarding devices throughout the system to ensure (1) that self-care behavior does not solely rely on personal initiative and (2) that such behavior is not penalized by management or assessment systems.

***4.1.2 Mitigating ethical risk through impact strategies***

Ethical risks can be mitigated through strategy, design, and planning at every stage of the research impact process. A key input to impact strategy is an analysis of the identities and needs of all relevant parties to help achieve effective and genuine collective representation ( Brugha & Varvasovszky, 2000; Reed *et al.*, 2009; Reed & Curzon, 2015). Moreover, a good impact strategy should help to ensure that research impact generation efforts do not inadvertently exacerbate social inequities or lead to opportunity costs for those who engage with little or no benefit ( Bærøe *et al.*, 2022; Broder *et al.*, 2024; Cooke & Kothari, 2001; Reed *et al.*, 2021).

Ethical impact strategies require at least some considerations of context, voice, and power ( Reed & Rudman, 2023). In particular:

- Context goes beyond biophysical, cultural, and geopolitical considerations to include the lived experiences and the socioeconomic and political contexts of people who might benefit from or suffer as a result of research, as well as their potentially contrasting world views and perspectives. To be ethical, researchers must consider these contexts and design research impact strategies accordingly in collaboration with these groups from the outset and embed them throughout the process.
- Inevitably, certain voices are heard and amplified in research processes. Researchers ought to include a wide range of perspectives and values in ways that authentically encourage those voices to be active in the research and research impacts.
- It is crucial to understand and manage the (often hidden) power dynamics and equity, diversity, and inclusion factors inherent in processes of impact generation (e.g., Broder *et al.*, 2024). This includes both overt “power over” and covert “power with” processes (e.g., how issues are framed for both research and impact). It is also essential to have an intersectional understanding of power rather than assuming that power relations are unidimensional.

Research impact strategies should reflect the purposes and the outcomes desired by (often conflicting or ambivalent) relevant parties. Inevitably, such design also reflects the ontology and epistemology of those who shape a project and its impact plan. Ideally, this shaping is done in collaboration between academic and non-academic research partners and explicitly accounts for all parties’ assumptions and beliefs about the problems and priorities they wish to address. Thus, researchers need to understand their positionality (per Alcoff, 1988, and others) in relation to both the research and its impact (e.g., Jackson *et al.*, 2023), including an awareness of biases and assumptions as these may influence the research impact process. The need for reflection and acknowledgement of positionality applies broadly ( Bilgen *et al.*, 2021) to how research is ideated and planned; how affected, interested, and influential individuals and groups are engaged with/involved; how knowledge, relational, behavioral, or policy change is implemented; and how such outcomes are monitored, evaluated, reported, and leveraged. Assessing and acknowledging positionality (e.g., Hauge, 2020; Martin *et al.*, 2022; Secules *et al.*, 2021) allows us to understand research impact in relation to contributors around the time of data capture, additionally outlining limitations of research and impact. At the same time, failure to understand, respond to, and account for positionality may lead to unintended consequences such as disempowerment, inaccurate assumptions, and flawed recommendations for policy and practice. For example, Global North researchers working to generate impact in the Global South may face distinctive ethical challenges when proposing or influencing change ( Collyer, 2018; Espig *et al.*, 2024; Moosavi, 2020; Williams, 2013).

### 4.2 Communication and engagement processes

We next focus on the role of ethics in the communication and engagement aspects of developing research impact. The National Coordinating Centre for Public Engagement in the UK defines engagement as “the myriad of ways in which the activity and benefits of higher education and research can be shared” ( National Co-ordinating Centre for Public Engagement, 2024). Generating impact typically requires some external engagement ( Bayley & Phipps, 2023), even though many impact pathways do not rely primarily on communication or engagement (e.g., patents, spinout companies, and analytic software; Jensen *et al.*, 2022a; Jensen *et al.*, 2022b). Many researchers believe there is an ethical obligation to communicate openly about their research and to respond to public concerns in a way that demonstrates humility, attention to accessibility, and respect to public audiences ( Royal Society, 2006). However, empirical studies show that audiences of public engagement activities tend to be considerably more highly educated and already interested in research than population averages ( Jensen *et al.*, 2021; Jensen *et al.*, 2022b; Kennedy *et al.*, 2018). This pattern of “preaching to the converted” raises questions about the inclusivity of research impact framed as public engagement. Similar concerns about the elite nature of engagement have been raised regarding researchers’ efforts to influence policy.

There are also ethical issues around how specialized research knowledge is communicated via news media and the like. In these settings, researchers’ commitment to accuracy must be balanced against other considerations. Medvecky and Leech (2019, p. 42) took this further to emphasize the ethics of time, place, and speaker, drawing on the Ancient Greek concept of *Kairos*: choosing the “right time, place, speaker, and audience is essential to an ethical moment of communication where people form beliefs […] The ethical ‘tinge’ to the concept comes from the ‘rightness’ of both what is communicated and the timing of that communication”. The tension between speed and rigor in public communication of research was brought into sharp focus by the COVID-19 pandemic. For example, there was increasing reliance on evidence from pre-prints (that had not undergone peer-review but saw unprecedented use by decision-makers during the crisis ( Majumder & Mandl, 2020), and an over-reliance on secondary sources such as press releases ( Spec & Schwartz, 2020). Today, new opportunities and challenges are emerging, including the use of Generative AI tools such as ChatGPT to help communicate effectively and efficiently (e.g., Institute for Methods Innovation, 2024). In fact, there have long been calls to clarify complex messages for public audiences rather than merely aiming to simplify. Another example comes from the field of public history, where debates centered on “whose history is it anyway?” reveal gaps between academics and non-specialist or community groups over how the past is communicated ( Ashton & Kean, 2009).

In addition to the need for timely access to information, the extent to which specialist knowledge can be understood is a critical consideration. The ethical issues arising from this tension play out in different ways depending on the communication methods used. For example, Dahlstrom and Ho (2012) point out that narrative and storytelling methods are often used in this context, which raises a set of ethical issues around the balance that is maintained between accuracy and accessibility of information in these methods. This challenge also applies to the creation of narrative scenarios, which are often used to communicate uncertainty to decision-makers. Participatory methods might help achieve an effective balance between accuracy and accessibility when communicating uncertainty to decision-makers. Similarly, infographics and data visualizations can convey information quickly and effectively, making it easier for non-specialists and others to understand complex patterns, relationships, and trends or quickly grasp new insights. However, visual communication methods can lead to biases in interpretation and misinformation or inadvertently play into negative stereotypes or prejudices (e.g., Jensen *et al.*, 2024). Choices around the scales used in graphs, maps, and other visualizations may be manipulated to convey biased points, and visualizations may privilege certain types of information (e.g., quantitative) over others (e.g., lived experiences) ( Monmonier, 2018; Reed, 2025). Hyped, biased, or misleading presentation of research results (e.g., exaggerating relevance or generalizability) can distort the potential understanding and decision-making of research users, leading to misguided policies or practices, misallocation of resources, and other adverse outcomes. Clearly, then, those communicating research findings ought to take into account ethical considerations of how their research outputs might be received, distributed, interpreted, and applied in different contexts. Having said this, the ethics of communicating research results extends beyond the researcher to issues and modes of wider dissemination; once published, such information has a life of its own and may travel through multiple, unpredictable itineraries.

Partly in response to these challenges, Antoni and Beer (2019) contend that the engagement and impact process should be understood as relational and emergent, not instrumental (see also Porter, 2018, regarding triple-rigorous scholarship: ethical, emotional, and epistemological). One increasingly influential example of relational and emergent approaches to engagement and impact can be drawn from Indigenous epistemologies ( Smith, 2021). Indigenous approaches to knowledge tend to be collectivist in nature, integrating engagement with the research process and valuing respect, reciprocity, and relationships ( Naepi, 2024). Those impacted by the research are included in the research design, processes for evaluative conversation are built into the implementation of the research, and communities remain custodians of the research beyond the project. The impact is enhanced (or made possible at all) because of the time taken to engage others in research processes from the outset (e.g., Polfus *et al.*, 2017). Indigenous approaches to knowledge offer different understandings of time, inviting relational and emergent approaches to engagement. An Indigenous approach to the ethics of impact is shaped by connections with generations past and is intended for children not yet born. Such an approach emphasizes capacity building and reciprocity, as evident in emerging guides and protocols for researchers (e.g., the Pacific research protocols from the University of Otago: Bennett *et al.*, 2013). More generally, a focus on the relational nature of impact highlights the importance of process in research impact, not merely the end results that are achieved. As Bayley and Phipps (2023, p. 8) note, “ethics includes considerations of the relationships between researchers and non-academic stakeholders and the values that underpin these endeavours”. From this perspective, impact work should be embedded throughout the research process, not viewed as something that happens only at the end when results are available (e.g., Reed, 2018; Reigersberg, 2011).

Participatory and co-productive processes (defined in Merkle *et al.*, 2022 with respect to communication of research) are ideal in communication and engagement because the creation of relevant and ethical impacts with research participants hinges on the processes of negotiating values, goals, and power dynamics ( Darby, 2017; Merkle *et al.*, 2022; Reed & Fazey, 2021). Ensuring that research impacts are both meaningful and ethically sound requires cultivating mutual understanding and balancing different interests and perspectives.

However, despite the broad support for co-production of research impact as an ethical practice, it is essential to acknowledge that enacting participatory research processes does have notable costs and downsides (e.g., Oliver *et al.*, 2019). Designing and implementing more participatory research impact processes does not guarantee more ethical outcomes. Well-meaning impact co-production can still be damaging in many ways (e.g., Cooke & Kothari, 2001), leading to elite capture ( Craney, 2020), exacerbation of conflicts ( Redpath *et al.*, 2015), and a loss of faith in participants. Researchers seeking to engage local communities may risk neglecting the specific cultural and social histories that are a part of what may motivate people to participate (or what is limiting their capacity to participate) in the first place (e.g., Polfus *et al.*, 2017; Razai *et al.*, 2021; Warren *et al.*, 2020). Co-production efforts can be used and/or perceived as a mechanism for consent, control, cooperation, accountability, and/or as a cynical technique for enhancing trust ( Ballesteros & Dickey-Collas, 2023). Some have called this the ‘tyranny’ of participation ( Cooke & Kothari, 2001).

Thus, to effectively and ethically engage others in and around research impacts, it is necessary to balance the needs for effective representation of interests and good process design with careful, skilled, and purposeful management of power dynamics, considering the values of participants, plural ontologies and their epistemologies, and the wider socioeconomic, cultural, and institutional context in which engagement is occurring ( Broder *et al.*, 2024; Dawson & Jensen, 2011; Reed, 2018). Additionally, in areas such as engineering or biotechnology, where research often seeks to advance research ideas from basic principles through technology readiness toward full system operation and even commercialization, it is vital to consider the broader enabling environment and simultaneously assess societal, policy, and industrial readiness levels ( Bernstein *et al.*, 2022; Francis *et al.*, 2023).

Furthermore, it is important to recognize the range of impact partners, especially when researchers work with other types of institutions, organizations, and communities. Such partners bring their own cultures and their many and varied imperatives ( Bilgen *et al.*, 2021; TRUST, 2018; Wagoner, 2017). Engaging non-academic actors can include working with nonprofit organizations, informal citizen groups, industry groups, and policymakers, who may have their own histories with marginalized communities. In some cases, such entities can serve as intermediaries with communities and nonprofit organizations that have long-standing community engagement initiatives. In other cases, relationships between impact partners and intended community beneficiaries may be strained or could even be characterized as harmful (e.g., police forces or international governmental organizations; Rosenbaum, 2002; Oliver *et al.*, 2019; Williams *et al.*, 2020). Ethical impact requires addressing such dynamics within a communication or engagement strategy to effectively and ethically address and navigate them.

Taking these factors into account, risk assessment and mitigation strategies may be required to foster inclusive and positive co-production processes and outcomes for communication or engagement. Goals should, wherever possible, be agreed upon collaboratively between researchers and all relevant parties upfront, and roles should be clearly defined. It is helpful to seek and deliver on short-term actions that feed into medium-term goals, to provide proof of integrity, and to help build trust between other actors and the researchers. Indeed, it is time to include facilitation, mediation, and communication skills as an explicit requirement for researchers involved in these projects (e.g., Broder *et al.*, 2024). Munshi *et al.* (2020) argue for the adoption of a culture-centered framework for public engagement to address shortcomings and injustices associated with top-down approaches to disseminating scientific information. At a minimum, research training requirements should include active listening, tools for cross-cultural engagement and conflict management, and setting up an openly available, shared vocabulary and goals framework at the project outset.

### 4.3 Evaluating and evidencing research impact

Just because researchers try to generate research impact does not mean that a positive outcome will always be successfully achieved. As we have discussed, many factors beyond the researcher’s control affect whether targeted research impacts come to fruition or fizzle out. Moreover, the foundations for impact may require years of investment of time, resources and relationship building from researchers ( Jensen & Gerber, 2020; Tsey *et al.*, 2019). Effective monitoring and evaluation processes can enable an evidence-based approach to improve the odds of success in developing research impact ( Jensen *et al.*, 2023).

Evaluation is a critical aspect of ethical research impact, as it provides accountability and can help identify and mitigate potentially problematic processes and outcomes (e.g., Jensen *et al.*, 2021; Oliver *et al.*, 2019). Specifically, evaluation incorporated from the beginning can provide “researchers with formative feedback that can enable them to learn from mistakes, identify and hopefully reduce negative outcomes during the pathway to impact, and build capacity for more responsible research and innovation” ( Reed *et al.*, 2021, p. 3). Further, the lens through which impact is evaluated may be an important factor in framing the research, its processes, and outcomes. For example, Chapman *et al.* (2020) advocate using the UN Sustainable Development Goals ( United Nations General Assembly, 2015) as a framework for evaluating impact based on the potential benefits of aligning research outcomes with these goals.

One ethical dimension of evaluating impact is ensuring that differentiated data are available to show whether there are any systematic patterns affecting particular social groups that are being engaged, for example, based on demographic variables such as gender, ethnic group and socio-economic status (e.g., UNECE, 2020). Measuring such variables can be a challenge in and of itself. However, there are options for borrowing from the measurement development work that has been done for national and international research projects by organizations such as the European Union, the OECD and the United Nations (e.g., Balestra & Fleischer, 2018; European Commission, 2020; Farkas, 2017). Such projects provide ready-to-use options for measuring variables such as racial or ethnic origin ( High Level Group on Non-discrimination, Equality and Diversity, 2021), gender and sexual orientation ( High Level Group on Non-discrimination, Equality and Diversity, 2023; National Academies of Sciences, 2022) and many other variables (e.g., European Social Survey, 2022) that can affect intersectional equality in research impact.

The complexity of establishing causal links between scholarly research and societal impacts is a major challenge, given the range of research outputs and confounding factors that may have contributed to an impact ( Greenhalgh *et al.*, 2016; Smith *et al.*, 2015). Given the subjectivity and pluralistic value judgments involved in evaluating whether an impact is beneficial or not ( Reed *et al.*, 2021), there is now a growing body of literature and practice on participatory monitoring and evaluation (e.g., Burns *et al.*, 2021; Onyango, 2018), building on a long history in development studies ( Guijt & Gaventa, 1998). Explicitly framing the context and purpose of the evaluation with both evaluation subjects/participants and evaluation end-users is a critical component of any responsible evaluation (e.g., Jensen, 2014; Jensen, 2015; Jensen, 2020; Jensen & Laurie, 2016; Jensen & Gray, 2025). Indeed, Antoni and Beer (2019) note that ethical evaluations should strengthen relationships (i.e., build social capital) and acknowledge the contributions of those who ultimately benefit from the research. For example, concerns have been identified around methods for collecting testimonial evidence to support impact claims in the UK’s REF ( Watermeyer, 2019), with Research England (2022, p. 35) expressing “ethical concerns around the way corroboration of impacts may have been made, particularly where data was gathered from vulnerable individuals and groups”.

Muller (2020) situates evaluation in the development of new public management practices in higher education, noting that the UK’s REF, international rankings, and other accountability structures or marketizing dynamics can create perverse incentives for unethical or problematic evaluation behavior ( Vitae, 2020). In some countries, such as Poland, this is described as a condition of “punctosis disease” (in Polish: “ *punktoza*”), that is, chasing evaluation-related points, “impact factors” and similar scores for papers and journals, and other “success criteria” that often undermines collaboration between researchers who are at the same time competing in various rankings and “parameterization”-related processes (see also Broder *et al.*, 2024 for a discussion of the negative pressure this prestige paradigm places on impacts efforts).

Indeed, institutional recognition of research impact typically requires demonstrable benefits within specific time frames, particularly in relation to large-scale research assessments such as the UK’s REF. This has made impact evaluation and evidence critical dimensions of research impact while sparking concerns about overly simplistic metrics (e.g., Donovan, 2019) and linear models and narratives of impact ( Crawford, 2020). Such metrics may be unethical in their effects on researchers’ well-being and impact practice once adopted. These metrics also incentivize brief, extractive, “parachute” research versus sustained, long-term, and co-produced research. There is a clear need for improved ethical norms and practices that prioritize the well-being and autonomy of affected people, as well as explicit consideration of the ethics of the evaluation of research impact. Broder *et al.* (2024) provide a robust framework for articulating these issues, identifying one’s level of influence, and mapping out possible actions to correct related problems within academia.

### 4.4 Assessment of existing professional guidance for ethical research impact

Through our narrative literature review and workshops, we scoped guidance available in the peer-reviewed literature, as well as grey literature and, in some cases, guidance not publicly available beyond institutions’ own guidelines. Table 1 summarizes the guidance found through this process. Although not exhaustive, Table 1 is the most comprehensive assessment of guidance on ethical research impact that we are aware of, to date. In it, we include guidelines, codes of ethics, and principles from multiple sources, covering a wide range of topics, including effective collaboration, ethical engagement, and the minimization of harm. We highlight therein the diversity of approaches found in the literature and emphasize the importance of context-specific adaptations. Table 1 thus serves as the basis for our recommendations discussed below.

**Table 1.** Overview of existing guidance on ethical research impact (thematically organized under the most relevant principle from Table 2).

Title	Description	Reference
**Principle 1. Build capability and capacity among those who may be affected by research impact activities to engage as equals.**		
Pacific research protocols from the University of Otago	The protocols include three values that are significant for the ethics of engagement and impact: • “5.1 Meaningful engagement between researchers and research participants requires developing, maintaining, and sustaining relationships that involve mutual trust” (p. 109). Specific suggestions include establishing advisory groups to help build trust through the engagement process and ensuring researchers are trained to “consult” effectively (p. 109). • “6.2 Reciprocity in research requires that knowledge gained through research will be used to benefit research participants and (where relevant) other people” (p. 110). A number of suggestions are made including building capacity and capability to extend reciprocity (e.g., via training including the opportunity to gain qualifications) and ensuring accessibility of findings to local communities. • “11.1 Capacity and capability building is critical to improving Pacific knowledge outcomes through research” (p. 112) as part of a commitment to the empowerment of local communities.	Bennett *et al.* (2013)
Being Manuhiri	This guide for researchers seeking to work with Māori groups is relevant to researchers working with Indigenous groups elsewhere. The research focused on how “non-indigenous scientists embrace the geographical, cultural, and social places they find themselves as manuhiri (guests)”. In this sense, being a guest is not limited to geographic location; it can also be applied to metaphorical and conceptual places, stemming from the knowledges associated with Indigenous peoples. That is, it can be applied to real, imagined, and conceptual spaces. Nine indicators can help non-Indigenous researchers navigate the co-production of knowledge and practices. The term “methodological sensitivities” invites an embodied collective responsiveness by researchers and their collaborators (and funders) to the knowledge systems, situations, aspirations, challenges, and invitations. Ten attributes, principles, and guidelines were identified for researchers to engage appropriately with Māori.	Landcare Research (2024)
The scientist abroad: Maximising research impact and effectiveness when working as a visiting scientist	This guide encourages visiting scientists to engage in specific activities to foster trust and effective collaboration with local people. This involves tailoring research to local issues, involving relevant actors, and respecting local contexts and ethics. It suggests that successful collaborations based on clear communication and genuine partnership are more likely to deliver lasting benefits and improved outcomes.	Chin *et al.* (2019)
Co-creation toolkit: A guidance on design, development, and implementation	The Co-Creation Toolkit offers comprehensive guidance for effectively integrating co-creation in industry-citizen collaborations. It outlines practical steps for engaging citizens and industry leaders to co-create solutions that meet user needs and address societal challenges. The toolkit covers phases of co-creation from planning and conducting workshops to evaluating outcomes, emphasizing the importance of user involvement, mutual learning, and adapting to both offline and online formats. It provides tools and methods for fostering creativity, structuring information, and understanding user needs, ensuring innovations are socially acceptable and desirable.	Kuhn *et al.* (2021)
Engaged research principles and good practices	Open access features strongly in research ethics but is less well discussed in relation to engagement and impact. This guide suggests that an “open innovation” model should be followed where solutions to societal problems are co-developed transparently with beneficiaries and other relevant parties, creating new relationships or collaborations as part of a broader “open innovation ecosystem” (p. 3). It goes on to argue that “responsible researchers deliver open, transparent, and ethical activities across the research and innovation life cycle, responding to feedback from those who have been engaged and involved” (p. 3). It includes an “engaged researcher checklist” which asks: “if the research is addressing a societal challenge or issue of public concern, has the research team engaged and involved those stakeholders most affected?”	IUA (2022)
Minimum quality standards and indicators in community engagement	Although developed for international development practice, UNICEF’s community engagement minimum standards are relevant to research engagement, especially in lower-income contexts. Minimum standards are organized around the principles of participation, empowerment and ownership, inclusion, two-way communication, adaptability, localization, and building on local capacity. Minimum standards are also provided for impact generation activities (“implementation”), which focus on informed design, planning and preparation, managing activities, monitoring, evaluation, and learning.	UNICEF (2020)
**Principle 2. Engage ethically with all relevant parties.**		
The TRUST code: A global code of conduct for equitable research partnerships	This guide aims to prevent “ethics dumping”, which it defines as “the practice of exporting unethical research practices to lower-income settings”, and includes a number of guidelines? relating to the ethics of engagement and impact: • The need to establish the local relevance of research in collaboration with local partners, arguing that “research that is not relevant in the location where it is undertaken imposes burdens without benefits” (p. 2). • Engagement with local communities in post-study impact evaluation, ensuring “their perspectives are fairly represented” (p. 2). • Dissemination of research findings to local communities “in a way that is meaningful, appropriate, and readily comprehended” (p. 2). • Where impacts arise from “traditional knowledge”, monetary and non-monetary benefits that might arise should be identified with a “culturally appropriate plan to share benefits…agreed to by all relevant stakeholders”. The planning process for benefit sharing needs to take into account “power and resource differentials…with sustained efforts to bring lower-capacity parties into the dialogue” (p. 2).	TRUST (2018)
Analyzing who is relevant to engage in decision-making processes by interests, influence, and impact: The 3i methodological framework	This tool enables researchers to identify who is most relevant to engage with, to generate impact in a strategic and inclusive way. It enhances traditional stakeholder analysis by adding “impact” to the existing “interest” and “influence” criteria. This approach aims to identify and prioritize all relevant parties, especially marginalized groups, in decision-making processes. It proposes a typology of eight types of relevant parties and suggests adapting engagement strategies based on their interests, influences, and potential impacts.	Reed *et al.* (2025)
Research with not about communities: Ethical guidance towards empowerment in collaborative research	This guide on community engagement with research (based on the TRUST Code below) encourages researchers to ask questions based on four values: • “Fairness: How are the communities meaningfully involved in discussions about the aims of the research, including why it is needed and who will benefit? • Honesty: Have all background details been shared and discussed with the community, including the funding situation and the intentions of the researchers?; What procedures will be used for two-way, open communication?; What procedures are in place to ensure understanding of research issues without being patronising?; What promises are being made to the community and can they be fulfilled? • Respect: How are community preferences for engagement strategies being discussed and acted upon?; Are the relevant community spokespersons or representatives being consulted?; Is permission from community elders/leaders or representatives needed for this consultation?; How are the research team familiarising themselves with local culture – including organizational structures, history, traditions, relationship with the environment, and sensitivities? • Care: How are local needs and the potential for capacity building being taken into account in development of the aims? Is due attention being paid to the impact of the study and the study team upon the participants, their families, the local community, and the environment?” (p. 19). It also provides guidance on: • Planning for dissemination of sensitive or controversial findings, especially with vulnerable groups, to avoid altering power dynamics within communities, exacerbating conflicts, or creating stigmatization or discrimination; and • The ethics of evaluating research impact, suggesting that in the context of community engagement, “research without any perceived benefits is unethical”, if communities have invested resources (including contributing their time or knowledge) in the research. It suggests communities should be involved in impact evaluations, given credit for their input, and with agency to define criteria for evaluating whether or not beneficial impacts have occurred (from their perspective, rather than merely the perspective of researchers or funders). Where adverse impacts are identified, the need for a transparent approach to resolving complaints is emphasized.	Chatfield *et al.* (2018)
Guidance ethics approach: An ethical dialogue about technology with perspective on actions	This framework for integrating ethical considerations into the development and use of technology emphasizes the interconnectedness of technology and society. It proposes a process that includes understanding the context of technology, engaging in dialogues about its effects and values with beneficiaries, and formulating actionable strategies for ethical implementation. This approach helps ensure technologies align with societal values, fostering responsible innovation and ethical technology integration.	Verbeek & Tijink (2020)
Integrated research toolkit	Integrated research “involves a diversity of people contributing to a project. These diverse contributions might be different knowledges, understandings of a problem, concepts, frameworks, data, methods, skills, or interpretations. They can come from a wide range of domains including the humanities, mātauranga Māori, government, law, industry, community, business, creative arts, as well as within the sciences” (section: What is integrated research?). In this sense, integration refers to bringing into research a diverse range of individuals and groups who might be affected by, interested in, or influential over the outcomes resulting from the research. The integrated research toolkit includes a selection of tools and associated frameworks that can be used to explore how to ensure the realization of beneficial impact and negate the realization of harmful impact.	Robson-Williams (2024)
Outcome mapping	OM is a methodology for planning and assessing project impact. “It has been developed with international development in mind and can also be applied to projects (or programmes) relating to research, communication, policy influence, and research uptake” (p. 1). OM is based on five key assumptions, including the following: • “People contribute to their own wellbeing; there are no passive beneficiaries. People’s wellbeing includes agency – the knowledge and power to play a role in creating, maintaining, assessing, or adjusting the actions that affect them and ecosystems on which life depends. People who have no influence over the programmes reaching them are not being helped” (p. 1) • “Differing yet equally valid perspectives will always coexist. Actors will interpret things depending on their particular stake in a situation. The ways in which these stakeholders are motivated and act may differ and may not be consistent or supportive of each other. Engaging the relevant actors while recognizing, reconciling, or managing their differing impetuses for involvement is a normal part of an intervention” (p. 1). • “Ecological, social, and economic resilience depend on interrelationships. Sustainable improvements in wellbeing involve influencing interconnected contributions from a variety of political, social, and economic actors. The engagement of these actors in appropriate interconnected patterns of behaviour is essential in building the capacity of stakeholders to maintain or adjust their contributions as conditions change, as needs emerge, and as the actors themselves evolve” (p. 1).	Earl *et al.* (2001)
What is good practice engagement and impact?	This guide builds on prior research to propose nine good practice principles for engagement and impact: • Understand your purpose and pursue impacts you find intrinsically motivating rather than allowing extrinsic incentives to drive your engagement • Understand your context so you can engage with empathy, inclusivity, and sensitivity • Where relevant, co-design your engagement and impact • Draw on robust and open evidence • Monitor, evaluate, learn, and be accountable • Build your skills and confidence and support each other in your engagement and impact • Consider and manage the ethics and risks of engagement and impact • Strategically plan and resource your engagement and impact • Understand and manage power dynamics and your own positionality	Reed (2023)
Principle 3. Manage risk and reduce the potential for harm.		
Guidance note: Potential misuse of research	This guidance recognizes that “some research…could be misused for unethical purposes [and] has the potential to harm humans, animals, or the environment” (p. 37). It provides guidance on ways of minimizing negative unintended impacts from research “by recognising risks in good time and taking the right precautions”.	European Commission (2021)
How to complete your ethics self-assessment for EU grants	Most research funders provide guidance around research that could be used to compromise national security, where it falls into the hands of criminals or terrorists. Horizon Europe also provides guidance on the potential misuse of research for unethical purposes. It identifies research activities particularly vulnerable to misuse, such as surveillance development and genetic profiling technologies. It provides questions to help identify potential unethical uses and mitigation measures, including changes to the research design, limiting dissemination, and working with ethics experts. This guideline is supplemented by instruction focused on “Identifying serious and complex ethics issues in EU-funded research”, as well as a series of notes with domain-specific guidelines related to the fields such as: dual use items; potential misuse of research results; focus exclusively on civil applications; research on refugees, asylum seekers, and migrants; data protection; ethics in social science and humanities; ethics in ethnography/anthropology; and ethics by design and ethics of use approaches for artificial intelligence.	European Commission (2019)
Trusted research: Guidance for academia	“Trusted research” is designed to protect intellectual property, sensitive research, people, and infrastructure from theft, manipulation, and exploitation, including by hostile actors. This guidance helps researchers protect their intellectual property and manage risks in international collaborations and cybersecurity. This includes questions to vet potential research partners, the use of legal frameworks and contracts, and compliance with export controls, for example, when exporting technologies overseas.	National Cyber Security Centre (2023)
Responsible research and innovation (RRI) self‐reflection tool	This tool asks researchers to consider the different values, interests, and ideals of the relevant parties they engage with, how they can prevent potentially harmful impacts on the public or the environment, and identify strategies for preventing adverse outcomes from their research (in the ethics section) and how to include people with different genders, ethnicities, classes, ages, routines, experience, or level of power (in the public engagement section).	RRI Tools (2024)
The SATORI CEN workshop agreement (CWA 17145)	Part 2 of the agreement is an ethical impact assessment framework designed to help researchers anticipate and ethically assess research and innovation’s social and environmental consequences. The approach combines ethics assessment, impact assessment, and technology assessment.	CEN (2017)
The TechEthos societal readiness tool	This tool helps researchers keep track of the societal readiness level of their research defined as “the degree to which a product can be trusted to fulfil its intended benefits within a real-world social setting while adhering to ethical principles, preventing adverse societal impacts, and being governed as needed by robust legal frameworks” (p. 1). It encourages researchers to consider the ethical implications of their research during design, implementation, and use, “allowing users to make their own judgements about how effectively their products prevent possible negative societal effects while delivering intended benefits” (p. 1).	Francis *et al.* (2023)
Ethical OS toolkit – a guide to anticipating the future impact of today’s technology	This toolkit is designed to help those developing technologies identify potential risks from unexpected uses and find ways of mitigating these risks. It includes a checklist of eight risk zones to help identify emerging areas of risk and social harm, alongside scenarios and future-proofing strategies.	Institute for the Future and Omidyar Network (2018)
SIENNA ethical guidance for research with potential for human enhancement	This guidance provides comprehensive recommendations for conducting research related to human enhancement technologies. It addresses key ethical issues such as autonomy, health and safety, fairness, equality, informed consent, and privacy. The guidance emphasizes a multidisciplinary approach and the importance of considering long-term societal impacts. It includes practical steps for ethical assessment and mitigation strategies, aiming to ensure that enhancement technologies are developed and applied responsibly and ethically.	SIENNA (2022)
Consequence scanning: An agile event for responsible innovators	Consequence scanning is typically used in product or technology development contexts, but can be applied to research impact. It asks three questions: “1. What are the intended and unintended consequences of this product or feature? 2. What are the positive consequences we want to focus on? 3. What are the consequences we want to mitigate?” (p. 9). Questions are typically answered in workshops by expert participants who are able to identify actions to mitigate risks, which can be monitored in a consequence scanning log.	Doteveryone (2019)
Principle 4. Seek to ensure equity, diversity, and inclusion in engagement and impact.		
EU code of practice on citizen-engagement for knowledge valorisation	The Code covers social inclusion, diversity, and gender equality, ensuring the engagement of all target groups and addressing barriers to participation.	European Commission (2024)
The inclusive design guide	This guide helps designers “be aware of the context and broader impact of any design and strive to effect a beneficial impact beyond the intended beneficiary of the design”. It includes guidance on designing for uncertainty and integrating accessibility activities and tools to help avoid harm and ensure positive impacts.	Inclusive Design Research Centre (2022)
Ethical research in fragile and conflict-affected contexts: guidelines for applicants	UKRI has guidance on ethical engagement and impact for researchers working in vulnerable contexts, including: • “Criteria 5: Research plan demonstrates systematic consideration of ethics during dissemination phase.” This includes the co-production of dissemination plans to ensure research is not used to disadvantage vulnerable groups or increase inequalities, ensuring dissemination of research protects and does not harm those who engage, and creating equitable benefits at different scales, including locally. • “Criteria 6: Research plan demonstrates systematic consideration of ethics during monitoring and evaluation of the research.” This emphasizes monitoring and evaluating both positive and negative, as well as intentional and unintentional, outcomes from research, ensuring ongoing risk assessment and mitigation. It includes the inclusion of “meaningful post-research evaluation to evaluate how ethics were addressed and to evaluate research impact” and underlines feeding back evaluation findings to affected groups. Although the guidance does not explicitly state whether evaluation should occur within or beyond project timeframes, it notes the need to explicitly plan for and fund the evaluation of engagement and impact.	UKRI (2021)
On intersecting modes of responsibility in Australia and Aotearoa New Zealand: A case for reimagining responsible innovation	Here, the authors advocate for an “emerging dialogue” concerning both anticipations of impact and inclusion of “others”. “Anticipation involves contemplation of the potential futures research and innovation can create… [while] also acknowledging inseparable connections to the past… Furthermore, it is crucial to reflect on who anticipates consequences of research and innovation and to what end.” They go on to state that: “Inclusion refers to incorporating diverse people, worldviews, values, and knowledges… Fostering inclusive practices within Australia and Aotearoa should then go beyond exercises in generic consultation and elicitation, which can manifest not only as inadequate but potentially extractive engagement practices… realising the potential of forms of inclusion that go beyond generic, or even extractive, engagements requires dedicated infrastructures and mechanisms to better support Indigenous- and minority-led research and innovation.”	Espig *et al.* (2024)
**Principle 5. Maintain accountability and evaluate engagement and impact.**		
Assessing value for money: The Oxford policy management approach	The Value for Money/Value for Investment approach “is intended to guide evaluators in combining multiple values and kinds of evidence, to help people make warranted evaluative judgements. It neither prescribes nor proscribes what values should be included, rather it positions evaluation as inclusive and impartial”. The associated 5ES framework includes the concept of “equity”, which involves exploring how fairly benefits are distributed and to what extent our work is reaching marginalized groups.	King *et al.* (2023)
Society of Professional Journalists code of ethics	This ethical code is comprised of four overarching principles: • Seek Truth and Report It (e.g., provide access to source material when it is relevant and appropriate) • Minimize Harm (e.g., consider the long-term implications of the extended reach and permanence of publication. Provide updated and more complete information as appropriate) • Act Independently (avoid conflicts of interest real or perceived. Disclose unavoidable conflicts) • Be Accountable and Transparent (e.g., respond quickly to questions about accuracy, clarity, and fairness)	Society of Professional Journalists (2014)
Science with impact: How to engage people, change practice, and influence policy	This book guides researchers in thinking more deliberately about choices they make during the research process that can lead to (positive or negative) societal impact. Choices include: what questions to ask, who to ask them with, where to do research (over- and under-researched communities), who should participate in data collection, who can evaluate research and for what purposes, and how and with whom to communicate about the results of the research. The book has free online flowcharts to help guide researchers through these questions.	Toomey (2024)
Guidelines for good practice in evaluation	This guidance focuses on the evaluation with a number of ethical principles relevant to the evaluation of impact including: • “Integrity: The practice of evaluation should demonstrate responsibility to participants according to agreed ethical principles and assure the veracity and validity of the findings. • Independence: Evaluations should be independent of vested interests and power differences. • Accessibility: Findings of evaluations should be available in the public domain and communicable to agreed audiences. • Trust: No evaluation can effectively proceed without trust, which needs to be developed and nurtured through agreed ethical procedures for conduct and reporting that are fair and just to all. • Equity: The conduct of evaluation should respect the perspectives and human dignity of all participants and stakeholders irrespective of their position in professional contexts or social structures. • Transparency: The principles underlying an evaluation, its approach, ethical practices, limitations, and uses should be made explicit to all stakeholders. • Diversity: Evaluation should respect cultural, gender, and age differences, and strive to include all relevant standpoints including those of the traditionally disenfranchised, marginalized, or hard to reach” (p. 1).	UK Evaluation Society (2003)
Responsible innovation self-check tool	The COMPASS tool promotes responsible innovation in small and medium-sized enterprises (SMEs) through a self-assessment framework. It features a multiple-choice questionnaire that evaluates practices across four management sections: company management, idea generation, development and testing, and market and impact. The tool helps SMEs understand and implement responsible innovation by offering actionable insights and benchmarking against peers. It emphasizes organizational learning and practical application, guiding users through responsible innovation dimensions and suggesting practical improvements based on their responses.	COMPASS (2019); Tharani *et al.* (2020)
Sharing science through shared values, goals, and stories	This tool provides a step-by-step process by which researchers can identify/acknowledge their own values and positionality, then work to identify values and goals shared with the communities or other partners with whom they seek to work. The step-by-step guidance offered by this tool is rare in communication and engagement around science (aka scicomm) and thus offers a useful resource for people working in those settings.	Merkle *et al.* (2022)
Knowledge exchange concordat	This includes the principle that knowledge exchange should be achieved by “working transparently and ethically” via published strategies that identify relevant goals and beneficiaries and “published mechanisms…to assure the ethical integrity and quality of…knowledge exchange” (no page number).	Universities UK (2020)
MULTI-ACT collective research impact framework	This framework provides a structured approach to evaluate and enhance the impact of multi-stakeholder health research initiatives. It introduces a multidimensional impact assessment model that integrates scientific excellence, economic performance, social impact, patient-reported outcomes, and mission effectiveness. The framework emphasizes engagement, particularly involving patients, to co-create research agendas and assess impact comprehensively. It includes tools such as the Master Scorecard for monitoring progress and a digital toolbox for managing engagement and data collection.	Zaratin *et al.*, 2022
AAL guidelines for ethics, data privacy, and security	These guidelines provide a comprehensive framework for ensuring ethical excellence in the development and deployment of digital solutions aimed at active and healthy aging. They integrate compliance with legal standards like GDPR and the Medical Device Regulation with a continuous ethical dialogue involving stakeholders. The guidelines cover phases from conceptualization to market entry, emphasizing user involvement, data protection, and the development of ethically robust technologies. They aim to address ethical challenges and enhance the acceptability and success of digital solutions by fostering trust and meeting high ethical standards.	Dantas *et al.* (2019)
European code of conduct for research integrity	Although mainly focused on research ethics, the Code includes principles such as “honesty in reporting and communicating research in a transparent, fair, full, and unbiased way”, “respect for society, ecosystems, cultural heritage, and the environment” and “accountability…for…wider societal impacts” (p. 5).	ALLEA (2023)
Ethical impact assessment	This guide to ethical impact analysis (EIA) has six steps: • Conduct an EIA threshold analysis • Formulate an EIA plan • Identify the ethical impacts • Evaluate the ethical impacts • Formulate and implement remedial actions • Review and audit the EIA outcomes	SATORI (2017)
Research impact privacy notice	A privacy notice that explains how Nottingham Trent University collects, stores, and uses evidence of the impact of its research. This could be adapted for use across the sector to increase transparency around impact data collection.	Nottingham Trent University (2023)
United States National Science Foundation	Grant reviewers will evaluate your Broader Impacts statement on these five criteria: • What is the potential for the proposed activity to benefit society or advance desired societal outcomes? • To what extent do the proposed activities suggest and explore creative, original, or potentially transformative concepts? • Is the plan for carrying out the proposed activities well-reasoned, well-organized, and based on sound rationale? Does the plan incorporate a mechanism to assess success? • How well qualified is the individual, team, or institution to conduct the proposed activities? • Are there adequate resources available to the principal investigator (either at the home institution or through collaborations) to carry out the proposed activities?	National Science Foundation (2024)
Principle 6. Design for lasting impact.		
Impact culture	This book identifies four components of a healthy impact culture, starting with the need to base impacts on rigorous, ethical, and action-oriented research and broadening to principles with associated actions around researcher motivations (or “priorities”), community building, and capacity building. Research and co-production: • Systematically prioritize stakeholders using stakeholder analysis • Pro-actively manage risks arising from impact • Practice open research • Make evidence synthesis more attractive and accessible Priorities • Engage researchers in a coaching process to identify forms of engagement and impact that they might find intrinsically motivating • Organize internal impact-related events that will engage researchers with varying levels of interest and experience with impact • Harness the power of your communications in creative new ways Community • Create a compassion culture • Experiment with more creative stakeholder engagement initiatives • Create boundary organizations • Co-produce events with your non-academic partners Capacity • Build skills for impact • Resource impact • Build your learning capacity • Do you need an impact strategy?	Reed (2022)
Ten principles of high-quality engagement	1. Understand your purpose and your context 2. Consider carefully the people you hope to involve in your engagement work and the role of equality, diversity, and inclusion in your approach 3. Design your approach with your purpose and people in mind, and where possible, involve others in the design phase 4. Use evaluation strategically, and make sure you use it to reflect on your work and with your team 5. Anticipate, explore, and manage the ethical implications of your work and ensure that you do no harm 6. Plan and resource your work appropriately, getting help where needed. Be sure you have expert administrative support 7. If you work in partnership with others, take good practice partnership principles into your work 8. Reflect on the power dynamics in your work and address these appropriately 9. Consider if and how you will sustain your work and manage the expectations of those involved 10. Work with others with relevant knowledge, networks, and expertise – this could be public engagement professionals within your institution or partnering organization	NCCPE (2023)
Analysis of public engagement with H₂ via social media channels across the EU27	This HYdrogen Public Opinion and acceptance (HYPOP) project report outlines strategic recommendations for aligning public engagement efforts with societal needs, including addressing ethical concerns that may emerge. These recommendations aim to tailor engagement strategies to specific regional interests and concerns, trying to ensure that messaging is both culturally and linguistically appropriate. Region-specific engagement strategies: • Develop strategies tailored to each region’s unique interests and concerns, utilizing data on topics that engage different populations. • Ensure engagement strategies are culturally and linguistically tailored to resonate with each community's context, improving the effectiveness of the messaging. Collaborate for unified messaging: • Partner with industry stakeholders, academia, and non-governmental organizations to create a unified message about the benefits and potential of the subject matter, amplifying the reach and impact of engagement efforts. Monitor and adapt strategies: • Continuously monitor public views, ensuring that engagement strategies maintain relevance and responsiveness to evolving public interests and concerns.	Institute for Methods Innovation (2024)
The societal readiness thinking tool: A practical resource for maturing the societal readiness of research projects	This tool helps researchers assess the societal readiness level of their research, asking reflective questions “intended to aid identification and accounting for key societal dimensions of innovation at different stages of a project” (p. 5). It is designed to complement Technology Readiness Levels by addressing broader societal concerns. The tool provides reflective questions at various stages of a project’s lifecycle, encouraging researchers to consider societal implications, engage with stakeholders, and adapt their work based on feedback. It promotes responsible research and innovation by facilitating early-stage identification of societal impacts and fostering continuous, iterative learning throughout the research process.	Bernstein *et al.* (2022)
Standard operating procedures for research integrity (SOPs4RI)	The SOPs4RI tool provides guidelines for promoting research integrity. It emphasizes developing, implementing, and maintaining a Research Integrity Promotion Plan (RIPP), which includes policies and procedures for fostering an ethical research environment. Key topics addressed in a RIPP include research environment, supervision and mentoring, research integrity training, data practices, research collaboration, publication and communication, declaration of interests, and handling breaches of integrity. The tool aims to create a supportive research culture by addressing issues like hyper-competition and promoting transparency, diversity, and inclusion.	SOPs4RI (2022)

## 5. Discussion: An integrated ethical framework for research engagement and impact

We advocate for ethically sound management of research impact. As researchers worldwide, we must fully consider the ethical issues surrounding research impact in addition to the well-established processes of research ethics. Both individual researchers seeking to make a difference, and research institutions that aim to support, enable, and embed ethical impactful research cultures and practices, and reduce the risks of research impacts, need to be engaged in this discussion. In this article, we emphasize the importance of pairing the pursuit of research impact with proportionate, ethical safeguards and with the resourcing and capacity-building required to adhere to them. A vigilant approach to ethics is particularly important in scenarios where economic gains might be prioritized, where marginalized, or disempowered communities are involved or affected, or when the potential for negative unintended impacts is high.

As described in Section 3, we conducted a narrative review of key sources of ethical guidance in the current literature and then refined these to develop an integrated framework for the ethics of engagement and impact for researchers and their institutions ( Table 2). The framework outlines principles and corresponding guidance for researchers and institutions to ensure ethical practices around the generation of impact. We pair each principle with specific actions that researchers should take, such as conducting needs assessments, co-designing research with impacted groups, and continued collaborative discussions across the impact lifecycle. Guidance for institutions includes the allocation of resources, training provision, and fostering a positive environment that supports ethical engagement and sustained impact.

**Table 2.** An integrated framework for the ethics of engagement and impact for researchers and their institutions.

Principle	Description	Guidance for researchers	Guidance for research institutions and funders
1. Build capability and capacity to engage as equals among those who may be affected by research impact activities.	Be led by the needs and priorities of those who may be affected by or interested in engaging with research impact. Build capability and capacity with these groups, paying attention to power dynamics, enabling them to engage (to the extent they so desire) as equals with researchers through knowledge sharing, access to resources, training, and other forms of support as appropriate to the context.	• Assess the capabilities and capacities of all relevant parties (including researchers and impact professionals within institutions) to determine needs, enabling these groups to lead the assessment themselves where possible and where they so desire. • Offer resources, training, and other opportunities that are tailored to the specific needs and contexts that have been identified in the needs assessment, where relevant, leading to qualifications. • Empower non-research partners to take on leadership roles within the project, providing them with the necessary resources and decision-making power to ensure the research delivers impacts that meet their needs.	• Consider resource allocation to relevant third parties to enable their meaningful engagement. • Reimburse institutions for 100% of the costs of engaging with non-research partners and make this a formal part of the project-costing process or cost into funding bids as part of impact activities (with implications here for funders whose rules may not currently allow this).
2. Engage ethically with all relevant parties.	Engage meaningfully with all relevant parties, including place-based communities, communities of practice, non-research partners, and other individuals and groups who may be interested or affected by the research outcomes.	• Systematically analyze the relative interest, influence, and impacts likely to arise for those who engage with or who might benefit from or be harmed in any way by the research, for example, using an interest-influence-impact (3i) analysis ( Reed *et al.*, 2025). • Establish culturally appropriate and accessible, two-way communication mechanisms with the relevant parties identified (e.g., via workshops or advisory groups), to ensure ongoing dialogue, and respect local knowledge, traditions, and cultural contexts. • Co-design research, where possible, with those who stand to benefit or lose most from its outcomes to ensure relevance and usefulness and reduced negative outcomes. • Critically analyze the power (im)balance inherent in how the research is framed and the extent to which the work can be driven by those it is engaging and/or intended to benefit (vs. researchers), including engagement and impact planning and project governance. • Avoid “ethics dumping” (the practice of researchers or organizations conducting research in countries or populations with less stringent ethical standards than their own, often exploiting vulnerable communities by applying lower ethical safeguards than would be permitted in their home country) by maintaining consistent ethical standards across all settings in which impacts may arise from research, particularly in lower-income or vulnerable communities. • Co-create an ethics charter with all relevant parties that outlines the ethical standards expected in the project, including research methods, engagement, and impact.	• Provide training and guidance on engagement for postgraduate and postdoctoral researchers, as well as for ECRs and all other staff with responsibility for research and/or engagement on an ongoing basis (including as part of induction processes). • Upskill research mentors and research leads to advise on best practice, identifying local champions. • Provide evidence-based best practice guidance to underpin training. • Create cohorts of local/civic community researchers, for example, via visiting community researcher schemes or funding community researchers to be trained and receive qualifications to work alongside researchers in the institution. See, for example, the University of Staffordshire’s (2024) Connected Communities team, the Scottish Institute for Policing Research’s (2024) Practitioner Fellowships, and the Vulnerability and Policing Futures Research Centre’s (2024) Translational Fellowships. • Advocate funders to allow researchers to build “flexible funds” for non-academic groups to apply for and use project funds in self-governed mini-projects that contribute towards the aims of the project and that are not overly burdensome to spend and account for. • Recruit (and appropriately compensate) non-academic ethics reviewers to evaluate applications that include especially high-risk engagement activities or demographics. • Embark on deeper culture change work to increase the value placed on ethical engagement by researchers and the need for them to invest in building their capacity in this area.
3. Manage risk and reduce the potential for harm.	Proactively identify, assess, and attempt to mitigate potential risks and negative impacts arising from research and engagement, both during and after the completion of research.	• Use interest-influence-impact analysis to identify at-risk groups, engaging with these and other relevant parties to identify potential risks and associated mitigation strategies. Working with these groups, the widest possible range of future scenarios should be identified in which the research could potentially create risks or cause harm, both during and after the completion of the research. • Plan for the monitoring of risks and harm during and after projects. Where possible, build this into funding proposals and consider the dynamics of impact over multiple time frames. • Monitor foreseen risks and harm while being alert to the possibility of new and emerging risks and harm throughout the project, with clear protocols for addressing issues as they arise. • Make all relevant parties aware of institutional complaints procedures so they can report risks and negative impacts. Alternatively, these procedures may be created and managed at the project level. • Use risk assessment to take into account uncertainties, support decision-making, and guide the research impact strategy.	• Enable researchers to quickly and easily identify engagement and impact plans that may be high risk, for example, via an online survey tool, giving automatic ethics approval to low-risk activities, conditional approval to medium-risk activities if researchers engage with relevant training and guidance, and referring the highest-risk activities to ethics committees. • Where necessary, provide access to ethics experts to help researchers adapt their research and engagement strategies to avoid and manage risk appropriately. • Enable monitoring of risks and harm after the completion of projects to ensure ethical interactions do not stop when project-based funding for this activity ends. • Establish mechanisms that enable local communities, non-academic partners, and other relevant parties to report risks or negative impacts they observe or experience. Moreover, make researchers aware of these processes so that they can be promoted to all relevant parties. • Organize periodic institutional learning exercises with key partners in order to meet with and respond to the experiences these partners have in engaging with researchers and the institution.
4. Seek to ensure equity, diversity, and inclusion (EDI) in engagement and impact.	Ensure equitable, diverse, and inclusive engagement by systematically assessing and addressing barriers to engagement by all relevant parties, with a particular focus on raising up the voices of those with the least power.	• Systematically consider and include diverse genders, ethnicities, ages, and other demographic factors in the engagement process to ensure that all voices are heard, valued, and considered equally. • Make deliberate efforts to proactively identify and remove barriers by taking appropriate actions, enabling everyone to engage equally. • Adapt engagement processes, communication channels, and approaches to be inclusive, accessible to, and meet the needs of different groups based on an analysis of their interests, influence, and impact (see Principle 2 above) • Develop actionable and measurable equity, diversity, and inclusion delivery plans.	• Embed EDI and engagement in research culture by developing policies and processes that enable and reward diversity and inclusivity in research projects, both pre-award and post-award, ensuring that they are reflected in every aspect of projects funded in the institution. • Provide necessary resources including internal funding and training to all team members on cultural awareness and the skills needed to respectfully navigate cultural diversity in different research settings.
5. Maintain accountability and evaluate engagement and impact.	Commit to accountability and continuous learning, engaging those affected by the research in evaluating engagement and impact, and using findings to enhance engagement and impact practice within and beyond the institution.	• Plan for evaluations of engagement and research impact, working with affected groups where relevant and appropriate to establish clear, measurable impact goals. • Evaluate engagement and impact with reference to relevant parties’ expectations, including assessing how ethical considerations were addressed. • Provide regular feedback on progress towards impact goals, challenges, and ultimate outcomes as they arise. • Where possible, involve independent evaluators and/or those affected by the research to assess the project’s impact.	• Provide ongoing support for researchers to evaluate their engagement and impact and learn lessons for their own practice. • Establish mechanisms to share lessons from evaluating engagement and impact across the institution, where possible, joining sector-wide initiatives to exchange learning. • Ensure the burden of evaluation is proportionate to the scale of engagement and impact. • Provide resources for evaluation, including independent evaluation, where this activity is not funded in projects and post-project evaluation.
6. Design for lasting impact.	Design research with a long-term perspective, aiming for lasting impacts where possible, maintaining flexibility to adapt to unforeseen barriers and opportunities, changes in the project’s context, and emerging ethical concerns.	• Integrate legacy planning into the project’s initial design. This should include plans for post-project maintenance, support, and funding. • Work with non-academic partners to develop their ability to continue the project’s initiatives after the research phase has ended (see Principle 1). • Plan for long-term studies to track the project’s impact years after its completion, adjusting strategies based on those findings to maximize long-term benefits. • Incorporate regular review points in the research process to assess the need for methodological adjustments or to address new ethical issues.	• Support adaptive research practices through flexible funding and project timelines, and establish mechanisms to respond quickly to necessary changes in research protocols. • Incentivize and support long-term engagement by researchers, with non-academic partners, between funded projects. • Dedicate funding to legacy initiatives, integrating across projects where possible to maximize impact and cost-effectiveness.

We have detailed extensive issues and necessary corrective actions in order to achieve a more ethical approach to research impacts. However, the momentum behind the policy push for “impact” and its measurement ( Oancea, 2019) is also contributing to increasing numbers of research professionals focused on impact. At the same time, researchers are allocating more time and resources to impact-focused work as a secondary or tertiary activity among job responsibilities more traditional to academia or research (e.g., teaching, administrative work in their institutions, and contributing to their professional societies) ( Jensen, 2020; Jensen & Holliman, 2016). In this context, there is both potential and an imperative to establish ethical standards to guide the training and practice of all researchers. The guidance we offer in Table 2 may require research institutions and funders to evaluate and possibly adapt their processes and structures across several domains. For example, academic institutions may need to adjust policies, procedures, and cultural attitudes in academic departments, institutional research offices, impact acceleration offices, institutional review boards, or research ethics committees.

A special urgency exists for training to minimize the risks and maximize the benefits of research conducted by early career researchers before problematic habits form and become embedded. Likewise, senior researchers will benefit from re-training. There are also essential considerations around managing risk to individual researchers and their institutions, since the burden of managing ethics is disproportionately put on their shoulders. Relatedly, the increased emphasis on impact work generates an implicit ethical responsibility for institutions to recognize, distribute the workload, and reward this kind of activity through appraisal and promotion structures and financial and human resourcing ( Broder *et al.*, 2024).

The first instinct for research institutions addressing the ethics of research impact may be to extend the umbrella of existing research ethics approval structures such as institutional review board (IRB) or “ethics committee” processes to encompass research impact. However, most IRBs explicitly exclude engagement and impact activities unless they involve the collection of personal data and lead to the generation of new knowledge (and are thereby classified as research). For example, the University of Edinburgh’s Guide to the Ethics of Knowledge Exchange (KE) Activities and Impact states that approval is only needed from the research ethics committee if KE or impact activity “involves any collection or analysis of data from human participants with the intent to answer research questions that will generate new and/or generalisable knowledge” ( University of Edinburgh, 2024). However, we highlight this particular guide because the University of Edinburgh’s ethics committee’s remit is wider than most, stating that approval is also required if participants may become individually identifiable or information is used in an academic or professional publication, whether or not this is classified as research.

In this context, it is essential to remember that bureaucratic processes can do more harm than good when they are disproportionate to risk, not well-resourced, and not implemented by people well-versed in ethical approaches to research impacts. There is a danger that a lack of impact expertise among members of IRBs may discourage impact planning instead of encouraging the mitigation of negative outcomes. Even if training was provided to board members, many IRB processes are primarily aimed at protecting institutions from legal liability, with ethical deliberation secondary or absent. Establishing systems within research institutions that are rigid enough to avoid unethical impact practices but flexible and lightweight enough to promote beneficial outcomes is very challenging.

A possible answer here is a “duty-based ethics” approach for relatively low-risk research impact scenarios. This could involve (1) triage to separate the low-risk from the high-risk activities, then (2) handling the low-risk activities in a way that focuses on capacity building and enabling good practice by the responsible parties. Meanwhile, high-risk activities should receive both capacity building treatment and a more bureaucratic, intensive review process similar to an IRB. To accomplish such triaging and subsequent resourcing, training would be needed for IRB members, professional services staff, and researchers, to distinguish low- and high-risk activities, build capacity for both, and provide meaningful support, accountability, and evaluation. Some consideration would need to be given to who does the triaging, and that this kind of work must be properly counted in workloads. For example, such triaging could be done by researchers themselves, supported by self-assessment questions to guide project team discussions about ethical impact. Questions could include: Are we confident that already marginalized, vulnerable, and/or disempowered individuals and groups will not be further harmed by this work? Have we taken reasonable efforts to identify and engage with such individuals and groups to help ensure a) that they will not be further harmed by this work, and b) we have maximized benefits relative to their needs, interests, and aspirations with the resources available for this project? However, self-assessment by researchers, even with clear guidance and training, could lead to varying interpretations of guidance and the improper categorization of risks. Specialist training for IRBs could provide more consistent and objective outcomes but would add to the burden of work on board members. Moreover, a focus on specialist evaluators could potentially exclude the perspectives of those likely to be affected by the research.

For both high- and low-risk scenarios, institutional expectations and duties for institutions and researchers should be made clear, with as low a burden as possible placed on those involved in impact activities under a certain risk threshold. Indeed, in some disciplines, the vast majority of impact activities may have little or no risk and need not trigger a formal process aside from the individual researcher ensuring that their approach is ethical and any risks are identified and managed. The need for an ethical framework to underpin this work does not mean that individual researchers should be dissuaded from seeking to generate impact. However, it is vital that risk assessment and accountability are coupled with incentives and institutional support to do so ethically.

 Research institutions also have a role to play in promoting and ensuring ethical best practices for impact. For example, research institutions may have internally managed funding programs, provide support for developing research impacts, offer training for researchers and professional services staff, and run initiatives enabling external partnerships. Research institutions also have internal processes for tenure and promotion that can be adapted to incentivize and support ethical research impact by providing guidance on how to credit non-traditional research products and processes. Some universities have already adopted such approaches by developing new metrics for the evaluation of community-engaged scholarship, thereby assisting with the assessment of research that involves extra-academic partners ( Toomey, 2024). Similarly, the “Hidden REF” evaluated and championed the inclusion of non-traditional outputs, including outputs co-produced with beneficiaries, for inclusion in future REF cycles.

 Research funders and those involved in higher education policy at national and international levels must also be involved in these conversations to ensure that incentive and administrative structures relating to research impact promote ethical practices. Indeed, many research-granting agencies are increasingly aware of the problematic nature of the “fund and forget” model and are seeking novel ways to accompany and support researchers in achieving extra-academic impact, For example, the Lenfest Ocean Program funds research projects that address the needs of marine and coastal communities and takes an active role in connecting scientists and decision-makers to ensure that the research results are accessible and usable.

## 6. Conclusion: challenging intersections between research ethics and impact ethics

As much as possible in this article, we have kept our focus trained on the ethical issues associated with generating research impact rather than the specific process of getting ethical approval to do research. The imperative to assess and manage the ethics of research is now more important than ever before, given the ethical arguments that research should benefit those who participate and the taxpayers who indirectly pay for it. Whether or not these normative arguments for impact are accepted, there is a growing recognition that ethical research can give rise to unethical engagement and impacts. Few research institutions and funders have processes to identify or manage the risks of unethical engagement and negative unintended consequences that lead to “grimpacts” ( Derrick *et al.*, 2018). This gap in professional practice is indicative of a gap in knowledge that this article has sought to fill by proposing an integrated framework for the ethics of engagement and impact.

 The implications of this work are far-reaching, given that many academic disciplines have not traditionally required formal ethics review processes related to human subjects, and few IRBs have adequate guidance or training to assess or manage the ethics of engagement and impact. For some disciplines, increased engagement with the impact agenda necessitates researchers who are unfamiliar with ethics review processes to embed such considerations into their research methodologies and practices. Similarly, STEM disciplines may benefit from a deeper consideration of the impacts of their work in science and technology development on human and non-human populations. This also applies to those working on more fundamental, basic research that does not require any direct interaction with, for example, humans, non-human animals, or the environment. That such research is often conducted by people untrained in research ethics, in human subjects ethics or, perhaps, even in social science methods, is a gap that needs to be addressed.

However, it is also relevant to consider the potential unintended negative consequences of these recommendations. For example, there is a danger that the introduction of new measures to assess the ethics of impact could undermine engagement activities and partnerships by adding a disproportionate administration load that could disincentivize impact-generation activities. Moreover, a new layer of ethics procedures relating to impact can be seen as an “audit culture” approach to mitigating organizational risk ( Oancea, 2019). Increased demands could disproportionately affect part-time, early career, disabled, neurodivergent, minoritized social groups, women, or researchers with caring responsibilities, as well as smaller, less-resourced institutions ( Watermeyer *et al.*, 2022). It is also necessary to consider the ethical implications of asking non-academic participants to collaborate when they already face significant demands on their time, especially in sectors like healthcare, education, and social work. The challenge lies in balancing the need for ethical practices with the feasibility of securing engagement, particularly for theoretical or exploratory research where benefits might not be immediately evident. Any implementation of the recommendations in this article should include strategies to mitigate impacts on marginalized groups, explore mechanisms to support under-resourced researchers and ensure equitable participation of non-academic partners. This comprises developing flexible and scalable ethical review processes that accommodate the diverse capacities and constraints of both academic and non-academic collaborators.

Our approach has several caveats. First, it is not based on a systematic literature review. Thus, some examples of guidance and related materials may have been overlooked during the exploratory process we have employed. Second, the selection of the ethical guidelines analyzed does not fully include non-English sources, and yet ethical research impacts must take into account other cultural paradigms. Third, while our gathering of interdisciplinary views was undertaken through online workshops and an open authorship model, such methods have different affordances than in-person approaches, and despite our extensive solicitation efforts, we likely did not reach all potential contributors. Fourth, by design, we excluded many related topics. Doing so enabled us to focus directly on research impact ethics but may also have implied analytical constructs, distinctions, or boundaries between topics that do not and should not exist. Finally, our work does not attempt to articulate a single, internationally applicable standard or regulation on ethical research impact. Though an effort toward such a standard would ideally be productive, doing so requires a scope of resourcing and international diplomacy that is beyond the remit of this article.

Notwithstanding these limitations, this article is the first international, interdisciplinary attempt to collate ethical guidelines relating to impact, distill principles and propose a framework that could guide the future practice of researchers, research institutions, and funders. The framework is applicable across disciplines and nations and should be relevant in a range of cultural and institutional contexts. It is hoped that this work will stimulate similar meta-research that could result in refinement and standardization of approaches to impact ethics and guide new, more ethically responsible research impact practices in research institutions and funding organizations internationally. By embedding ethical guidelines into the fabric of research institutions, the goal is to foster a research culture that prioritizes societal benefits while minimizing harm.

## Data Availability

No data are associated with this article.
