# Supplementary material for: Responsible research impact: Ethics for making a difference
Source: Open Res Eur. 2025 Mar 31;5:92. [Version 1] doi: 10.12688/openreseurope.19649.1 (PMC12181762; doi:10.12688/openreseurope.19649.1)
Supplement: Peer review history

# Reviewer report: Reviewer response for version 1

**DOI:** 10.21956/openreseurope.21255.r52686

**Published:** 2025-06-20

**Authors:** Katherine Yaw

**Affiliations:** 1 University of South Florida, Tampa, Florida, USA

This paper presents a framework for research impact ethics informed by both existing literature and collaborative online workshops. The authors do a strong job of motivating the need to focus on impact ethics and then providing recommendations that apply across research disciplines. The main area that I would recommend more information is in the methods for this project. There is relatively little detail about how you conducted the two parts of this study (see comments below). In addition to the methods, there are some other areas that could benefit from additional information or clarification. Please find comments organized by specific manuscript sections below:

 Introduction:

- p. 4, end of para. 1: By framing the ethicality of research impact in a binary way (i.e., ethical/unethical), it may suggest to readers that there is a clear line between these two. It may be helpful instead to think about this along a continuum, where impacts that are clearly (un)ethical occupy the endpoints. In the middle are impacts that may vary in their level of ethicality depending contextual factors, researcher intent, etc. In the context of researcher decision-making, this grey zone has been described as questionable research practices (QRPs; see e.g., Fanelli, 2009). You mention on p. 6 that QRPs are outside of the scope of the current project, which is reasonable. However, I suspect that this concept of an ethical grey zone could also apply to promoting responsible research impact by providing researchers opportunities to work towards more ethical research impact practices and outcomes. Essentially, it builds room for nuance into the conversation.
- Aside from the previous comment, I found that this section presented a clear, compelling case for the need to focus on ethical dimensions of research impact. I appreciated your emphasis on involving stakeholders beyond the researchers themselves in this endeavor.

Methods:

- This section provides a summary of the methods that were employed for both the narrative literature review and online workshops. While the details of these methods may not be expected in an Open Letter format publication, some readers may want to know more specifics about what you did, how you did it, and why you made these choices.
  - For the narrative review, how did you determine which literature to consult? Is there a comprehensive list of your sources somewhere? Table 1 cites a wide range of sources – is this the extent of the literature included? If so, it may help to state this somewhere. If there’s more, perhaps there could be a supplementary file or appendix that lists these for interested readers.
  - For the online workshops, how were the co-authors identified from among the >300 participants in the workshops? What else is known about the participants aside from their geographic location (e.g., professional roles, field/disciplinary background)? How were the interactions/discussions organized (e.g., small breakout groups, groups led by one of the co-authors)? Are the workshop materials publicly available somewhere? I would like to know more about what happened during these workshops and who the participants were.

Research impact ethics scope:

- As a reader, I found this section effective at framing and justifying the focus of this project. The emphasis on reducing the risk of unethical research impact work makes sense as a priority, and your acknowledgement of the opportunity for future work to expand into positive, proactive steps is helpful to research community members seeking to engage in more work on the ethical aspects of research impact.

Results: Key aspects of research impact ethics

- p. 6, end of first paragraph in section: “… to consider perceptions of impact processes and results,…” Whose perceptions are you talking about here?
- p. 7, paragraph about Antoni & Beer (2019): Add the closing parenthesis for the description of “victims” at the end of the paragraph.
- p. 7, paragraph starting with Darby (2017): In the quote from Reed & Fazey (2021), what is the difference in meaning between “for what purpose, and why” when it comes to knowledge generation?
- p. 7, paragraph starting “in addition to considering the use of participatory methods…”: This point about impacts being a mixture of beneficial and harmful is really important. I think this relates to my earlier comment about framing ethics along a continuum, as studies with these mixed outcomes may fall into that grey zone.
- p. 8, section 4.1.2: You mention the importance of positionality here. Somewhere in this manuscript, it would be helpful to know more about the positionality of this team of co-authors – who you are and what perspectives you bring to this particular discussion. (My "partly" response above to the question about adequately referencing differing views and opinions relates to the paper not providing any background information about who the people involved in this study are - both on the author side and the workshop participant side. It would help to clarify whose views have contributed to this study.)
- p. 9, paragraph starting “Participatory and co-productive processes”: You point readers to Merkle et al. (2022) for a definition – consider briefly defining these here as well.
- p. 10, paragraph starting “Furthermore…”: The final sentence seems a bit circular. “Ethical impact requires addressing such dynamics…to effectively and ethically address and navigate them [the dynamics?]”
- p. 11, end of paragraph 1: Consider defining REF for readers unfamiliar with this.

Tables 1 and 2: Your consolidation of ethical impact guidance here is a great resource for researchers across a broad range of contexts, as is your six principle integrative framework. I find the guidance for researchers and institutions/funders to be particularly helpful. As I was reading this paper, I was thinking about the challenges of producing a non-field-specific framework like this. Moving forward, I would be interested to see examples of how this framework can be (will have been?) applied to different disciplines or research contexts.

 Conclusion:

- p. 26, paragraph starting “The implications of this work…”: You may want to provide more context for the statement “many academic disciplines have not traditionally required formal ethics review processes related to human subjects”. In the US context, for instance, even social science research has been subject to IRB ethical review for the past few decades. This is just one Global North context, but for readers who might assume this is the norm, it would help to explain your claim a bit more.

Overall, I thank the authors for your efforts on this manuscript, and I hope that the comments above help to strengthen your presentation of this study. This is valuable work, and I look forward to seeing the impacts of this study!

Where applicable, are recommendations and next steps explained clearly for others to follow? (Please consider whether others in the research community would be able to implement guidelines or recommendations and/or constructively engage in the debate)

Yes

Does the article adequately reference differing views and opinions?

Partly

Are all factual statements correct, and are statements and arguments made adequately supported by citations?

Yes

Is the rationale for the Open Letter provided in sufficient detail? (Please consider whether existing challenges in the field are outlined clearly and whether the purpose of the letter is explained)

Yes

Is the Open Letter written in accessible language? (Please consider whether all subject-specific terms, concepts and abbreviations are explained)

Yes

Reviewer Expertise:

Applied linguistics, quantitative research ethics

I confirm that I have read this submission and believe that I have an appropriate level of expertise to confirm that it is of an acceptable scientific standard, however I have significant reservations, as outlined above.

# Reviewer report: Reviewer response for version 1

**DOI:** 10.21956/openreseurope.21255.r52682

**Published:** 2025-04-23

**Authors:** Cristian Timmermann

**Affiliations:** 1 University of Augsburg, Augsburg, Germany

The authors offer a quite extensive study on the ethical issues of research impact using a broad range of perspectives.

 I have only two smaller comments:

 There is a rather brief remark on “There is also rising consensus that it is critical to ensure that the benefits of research are shared with all segments of society, not just a privileged few” With a reference to UNESCO. While this is idea is repeatedly taken up – and rightly so – the authors could have gone somewhat deeper into it. There are few authors dealing with the “right to benefit from scientific advancement” defending such a reading of the human rights declaration (see for instance the work of Audrey Chapman, Jessica Wyndham, Yvonne Donders, and others), which I also consider highly relevant for discussing research impact.

 The article is in my opinion somewhat too hard on institutional review boards. I would suggest the authors to sharpen their criticism in this aspect somewhat more. While the authors specify the importance of ethics training for IRB members, which is a good point, the part of bureaucratization is somewhat too general. Remarks like “…many IRB processes are primarily aimed at protecting institutions from legal liability…” (p. 24) are quite unfair where many people devote regularly without compensation dozens of hours in reviewing and assessing proposals.

Where applicable, are recommendations and next steps explained clearly for others to follow? (Please consider whether others in the research community would be able to implement guidelines or recommendations and/or constructively engage in the debate)

Yes

Does the article adequately reference differing views and opinions?

Yes

Are all factual statements correct, and are statements and arguments made adequately supported by citations?

Yes

Is the rationale for the Open Letter provided in sufficient detail? (Please consider whether existing challenges in the field are outlined clearly and whether the purpose of the letter is explained)

Yes

Is the Open Letter written in accessible language? (Please consider whether all subject-specific terms, concepts and abbreviations are explained)

Yes

Reviewer Expertise:

applied philosophy

I confirm that I have read this submission and believe that I have an appropriate level of expertise to confirm that it is of an acceptable scientific standard.

# Reviewer report: Reviewer response for version 1

**DOI:** 10.21956/openreseurope.21255.r52679

**Published:** 2025-04-17

**Authors:** Tula Brannelly

**Affiliations:** 1 Auckland University of Technology, Auckland, New Zealand

Many thanks for the opportunity to review this important work. This is an ambitious piece of work that aims to provide guidance globally for researchers. These comments intend to strengthen the main focus of the work. 

 Regarding who needs an ethical framework for impact, does this also extend to funders and reviewers? For example, who is involved in designing impact and how impacts can evolve throughout the research. 

 Considering indigenous voices, are there particular groups with whom impactful research needs more careful consideration to avoid any unintended harms from research practices.  Was this a focus with the groups in Australia and New Zealand, for example. Was the participation adequate for community membership - is it possible to say a bit more about whose ethics this project speaks to?

 Excellent to see relationally as an aspect of ethical practice.

 The framing of the topics (3) talks to the power of the research team empowering marginalised people - this is not the reality of the situation.  The topics miss the opportunity to embed the end users of research as central to the design and conduct of research and the centrality of lived experience as an important knowledge base from which to build research and therefore impacts.  

 Section 4 - this assumption of positive (if any) impact is driven by academic and funder priorities and not by the community. The experience of research by some groups may be negative, but little attention is given to that experiential impact, for example. The question about this again, is from whose perspective are these decision made and who is involved.

 Examples of grim pacts would make this more accessible. 

 Section 4.1 - who is involved in this and what constitutes impact from whose perspective? Can impacts emerge during research to provide an opportunity for end users to guide and develop impacts? Is there space to consider legacies of involvement in research projects for people with extensive experience of research participation and how are communities worked with to understand how research is experienced over time?  

 Some key citations missing from the ethics of care section. More than receptive, lived experience shapes research in care ethics as a central tenet. 

 Do no harm is a reductionist position to take and an inadequate standard to set. No harm to a research team without lived experience is likely to be quite different to an inclusive team so this does not adequately safeguard people who join projects. Perhaps a more fitting aim is to have research that takes the time to directly benefit and be a positive experience for participants. The 'highly resource intensive' activity would be needed to ensure that research is beneficial and serves the community. Legacy thinking would help here to consider who comes before and after, and what impacts are left.

 Perhaps we can see that researchers are connected to their topics as they are personal and resonate in some way - and that we are emerging in our identities as researchers as we research and that learning can happen with the communities we research with. 

 Beyond positionally, it is also useful to consider motivations for being involved in research - what needs to change may be very differently understood. 

 Distinctive ethical challenges from Global North to Global south include colonising approaches which needs to be visible.

 4.2 Useful to refer to the ethics of AI here. If discussing indigenous ethics, it is useful to discuss intellectual property and impacts - see Te Ara Tika, for example. These actions need good relationships. 

 Perhaps the discussion and conclusions could be informed using the suggested further resources if considered key for this work.  What is considered high or low risk, for example, will differ depending on whose perspective counts.

Where applicable, are recommendations and next steps explained clearly for others to follow? (Please consider whether others in the research community would be able to implement guidelines or recommendations and/or constructively engage in the debate)

Partly

Does the article adequately reference differing views and opinions?

Partly

Are all factual statements correct, and are statements and arguments made adequately supported by citations?

No

Is the rationale for the Open Letter provided in sufficient detail? (Please consider whether existing challenges in the field are outlined clearly and whether the purpose of the letter is explained)

Yes

Is the Open Letter written in accessible language? (Please consider whether all subject-specific terms, concepts and abbreviations are explained)

Yes

Reviewer Expertise:

Ethics of care

I confirm that I have read this submission and believe that I have an appropriate level of expertise to confirm that it is of an acceptable scientific standard, however I have significant reservations, as outlined above.

## References

[1] : The ethics of care and transformational research practices in Aotearoa New Zealand. *Qualitative Research* .2017;17(3) : 10.1177/1468794117698916 340-350 10.1177/1468794117698916
